# Influence of Lamb Wave Anisotropy on Detection of Water-to-Ice Phase Transition

**DOI:** 10.3390/s24247969

**Published:** 2024-12-13

**Authors:** Andrey Smirnov, Vladimir Anisimkin, Nikita Ageykin, Elizaveta Datsuk, Iren Kuznetsova

**Affiliations:** Kotelnikov Institute of Radio Engineering and Electronics of RAS, Moscow 125009, Russia; andre-smirnov-v@yandex.ru (A.S.); anis@cplire.ru (V.A.); ageykin_niki@mail.ru (N.A.); datsuk.elizaveta@yandex.ru (E.D.)

**Keywords:** plate acoustic wave, piezoelectric plates, water-to-ice phase transition, wave anisotropy, wave attenuation

## Abstract

An important technical task is to develop methods for recording the phase transitions of water to ice. At present, many sensors based on various types of acoustic waves are suggested for solving this challenge. This paper focuses on the theoretical and experimental study of the effect of water-to-ice phase transition on the properties of Lamb and quasi shear horizontal (QSH) acoustic waves of a higher order propagating in different directions in piezoelectric plates with strong anisotropy. Y-cut *LiNbO*_3_, 128Y-cut *LiNbO*_3_, and 36Y-cut *LiTaO*_3_ plates with a thickness of 500 μm and 350 μm were used as piezoelectric substrates. It was shown that the amplitude of the waves under study can decrease, increase, or remain relatively stable due to the water-to-ice phase transition, depending on the propagation direction and mode order. The greatest decrease in amplitude (42.1 dB) due to glaciation occurred for Lamb waves with a frequency of 40.53 MHz and propagating in the YX+30° *LiNbO*_3_ plate. The smallest change in the amplitude (0.9 dB) due to glaciation was observed for QSH waves at 56.5 MHz propagating in the YX+60° *LiNbO*_3_ plate. Additionally, it was also found that, in the YX+30° *LiNbO*_3_ plate, the water-to-ice transition results in the complete absorption of all acoustic waves within the specified frequency range (10–60 MHz), with the exception of one. The phase velocities, electromechanical coupling coefficients, elastic polarizations, and attenuation of the waves under study were calculated. The structures “air–piezoelectric plate–air”, “air–piezoelectric plate–liquid”, and “air–piezoelectric plate–ice” were considered. The results obtained can be used to develop methods for detecting ice formation and measuring its parameters.

## 1. Introduction

The transition of water from liquid to solid states when the temperature drops below zero degrees Celsius is a first-order phase transition [1]. These transitions are accompanied by the release of latent heat. The effects of lowering the temperature of water on its structure and the structure of the formed ice have been actively studied theoretically using molecular dynamics simulations [2,3,4], Gibbs free-energy calculations [5], transition potential approaches [6], and first-principles calculations [7]. The fundamental properties of matter during this transition have also been investigated experimentally. The properties of water in critical and supercritical conditions have been determined [8,9,10,11,12]. Recently, an analysis of the thermodynamic process during liquid freezing has been carried out [13]. The effects of impurities on the liquid-to-ice transition have been investigated [14,15,16,17]. Currently, research on the water–ice transition in porous materials [18,19], food [20,21], and biological tissues [22] is ongoing. In all these studies, a variety of experimental techniques were employed, including Raman spectroscopy [14,17], X-ray analysis [11,22], and scanning calorimetry [12], among others. Each of these techniques has contributed to the understanding of this important physical phenomenon.

It should be noted that the issue of controlling such transitions is important not only from a fundamental, but also from a practical point of view. Ice formation is a dangerous process in the operation of highways, airplanes, ships, etc. [23]. This process can lead to the incorrect functioning of wind turbines [24] or aircraft wings [25,26]. Additionally, an important aspect is the control of the freezing process of food or biological tissues [27].

In recent years, icing sensors have been developed using several different physical principles. These principles include optical [28], resistance [29], admittance [30], capacitive [31], electro-optical [32], fiber-optic [33], radio frequency [34], and ultrasonic [25,26,27,35,36,37,38,39,40,41,42,43,44,45,46,47,48,49,50,51,52,53] methods. The acoustic sensors are of great interest and can be based on various types of acoustic waves. For example, bulk acoustic waves (BAW) [25,26,27,35,45], surface acoustic waves (SAW) [36,37,38,41,42,46,47,52,53], Love waves [40,43,48], and waves in plates [39,44,49]. It has been shown that the use of ultrasonic methods makes it possible to determine ice thickness [35], ice roughness [26], ice type [50], and the presence of a water film on ice surfaces [51].

Previously, it has been shown that the sensitivity to water-to-ice phase transition of higher-order Lamb and QSH waves propagating in some definite propagation directions in various piezoelectric plates (YZ-*LiNbO*_3_, YZ+90°-*LiNbO*_3_, ST,X-quartz, ST,X+90°-quartz, 36°YX-*LiTaO*_3_, or 36°YX+90°-*LiTaO*_3_) can increase or decrease, depending on the wave order, wavelength, and normalized plate thickness *h*/*λ* (*h*—thickness, *λ*—wavelength) [44]. As is known, the properties of acoustic waves (phase and group velocities, attenuation, electromechanical coupling coefficient, etc.) strongly depend on the direction of their propagation, i.e., on the anisotropy of the material. Therefore, the response of these waves to surface loading will also depend on the anisotropy of the material. It has recently been shown that, due to the anisotropy of higher-order Lamb waves in piezoelectric plates, it is possible to identify various liquids without the need for sensor films [54,55,56]. It was assumed that the anisotropy of these waves would also lead to their different reactions to the process of glaciation occurring on the surface of the piezoelectric plate. It should be noted that no such studies have been carried out before.

The aim of this work is the theoretical and experimental study of the effect of water–ice phase transitions on the properties of higher-order Lamb and QSH waves propagating in various directions through anisotropic piezoelectric plates.

## 2. Materials and Methods

### 2.1. Theoretical Method

**Figure 1:**
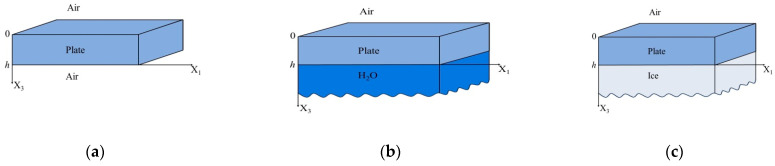
The geometry of the structures considered theoretically: (**a**) “air–piezoelectric plate–air”, (**b**) “air–piezoelectric plate–distilled water”, and (**c**) “air–piezoelectric plate–ice”.

For theoretical analysis, three structures “air–piezoelectric plate–air” (a), “air–piezoelectric plate–distilled water” (b), and “air–piezoelectric plate–ice”(c) were considered (Figure 1). For all structures, the acoustic wave propagation was taken along the x_1_ direction. The piezoelectric plate was bounded by the planes *x*_3_ = 0 and *x*_3_ = *h*. The regions with *x*_3_ > *h* corresponded to air (Figure 1a), distilled water (Figure 1b), or ice (Figure 1c), and the region with *x*_3_ < 0 always corresponded to air. The problem considered is two-dimensional, so all field components were assumed to be constant in the *x*_2_ direction [57].

The phase velocity, attenuation, and mechanical displacements of the acoustic waves could be found by using a system of appropriate equations for each medium contacted.

For the description of acoustic waves propagating in a piezoelectric plate, the motion Equation (1), Laplace’s Equation (2), and constitutive Equations (3) and (4) were used:

(1) $$\rho^{pz}\partial^{2}U_{i}^{pz}/\partial t^{2}=\partial T_{ij}^{pz}/\partial x_{j},$$

(2) $$\partial D_{j}^{pz}/\partial x_{j}=0,$$

(3) $$T_{ij}^{pz}=C_{ijkl}^{pz}\partial U_{l}^{pz}/\partial x_{k}+e_{kij}^{pz}\partial \Phi^{pz}/\partial x_{k},$$

(4) $$D_{j}^{pz}=-\varepsilon_{jk}^{pz}\partial \Phi^{pz}/\partial x_{k}+e_{jlk}^{pz}\partial U_{l}^{pz}/\partial x_{k},$$

For the description of acoustic waves propagating in nonpiezoelectric isotropic ice, the corresponding motion Equation (5), Laplace’s Equation (6), and constitutive Equations (7) and (8) were written:

(5) $$\rho^{ice}\partial^{2}U_{i}^{ice}/\partial t^{2}=\partial T_{ij}^{ice}/\partial x_{j},$$

(6) $$\partial D_{j}^{ice}/\partial x_{j}=0,$$

(7) $$T_{ij}^{ice}=C_{ijkl}^{ice}\partial U_{l}^{ice}/\partial x_{k},$$

(8) $$D_{j}^{ice}=-\varepsilon_{jk}^{ice}\partial \Phi^{ice}/\partial x_{k}.$$

Let us consider liquid as an isotropic solid media [58]. In this case, the motion Equation (9), Laplace’s Equation (10), and constitutive Equations (11) and (12) were written together:

(9) $$\rho^{lq}\partial^{2}U_{i}^{lq}/\partial t^{2}=\partial T_{ij}^{lq}/\partial x_{j},$$

(10) $$\partial D_{j}^{lq}/\partial x_{j}=0,$$

(11) $$T_{ij}^{lq}=C_{ijkl}^{lq}\partial U_{l}^{lq}/\partial x_{k},$$

(12) $$D_{j}^{lq}=-\varepsilon_{jk}^{lq}\partial \Phi^{lq}/\partial x_{k}.$$

Here, *U_i_*, *t*, *T_ij_*, *x_j_*, *D_j_*, Φ, and *ρ* are the components of mechanical displacement of particles, time, components of mechanical stress, coordinates, components of electrical displacement, electrical potential, and density, respectively. *C_ijkl_*, *e_ikl_*, and *ε_jk_* are the elastic, piezoelectric, and dielectric constants, respectively. The indexes *pz*, *ice*, and *lq* correspond to the piezoelectric film, ice, and non-viscous, non-conductive water (*H*_2_*O*), respectively.

In the regions of air, the electrical displacement is satisfied to Laplace’s equation:

(13) $$\partial D_{j}^{air}/\partial x_{j}=0,$$

where $D_{j}^{air}=-\varepsilon_{0}\partial \Phi^{air}/\partial x_{k}$. Here, *ε*_0_ is the air permittivity and the index *air* indicates that the variable refers to air.

In solving this problem, the quasi-static approximation condition for each medium was used:

(14) $$E_{j}=-\partial \Phi^{pz,ice,lq,air}/\partial x_{j}.$$

Here, *E_j_* is a component of the electric field strength vector.

The solution was sought in the form of plane inhomogeneous waves propagating along the *x*_1_-axis with an unknown distribution of amplitudes along the *x*_3_-axis in each contacting media [58].

(15) $$Y_{j}\left(x_{1},x_{3},t\right)\sim e^{i\omega t}e^{-i\omega x_{1}/V}e^{\omega \zeta x_{3}/V}.$$

Here, *V*, *ω*, and *ζ* are the phase velocity, the circular frequency of the acoustic wave, and its eigenvalue, respectively. *Y_j_* are normalized variables with dimension N/m^2^ corresponding to the components of mechanical displacement (*U*_1_, *U*_2_, *U*_3_), normal components of mechanical stress (*T*_13_, *T*_23_, *T*_33_), the electrical potential (Φ), and the normal component of electrical induction (*D*_3_) for each of the contacting media. Normalization for all media was performed using the material parameters of a piezoelectric material.

(16) $$Y_{1}^{pz}=\frac{\omega }{V}C_{1111}^{pz}U_{1}^{pz};\;Y_{2}^{pz}=\frac{\omega }{V}C_{1111}^{pz}U_{2}^{pz};\;Y_{3}^{pz}=\frac{\omega }{V}C_{1111}^{pz}U_{3}^{pz};Y_{4}^{pz}=T_{13}^{pz};\;Y_{5}^{pz}=T_{23}^{pz};\;Y_{6}^{pz}=T_{33}^{pz}$$

(17) $$Y_{7}^{pz}=\frac{\omega }{V}e^{pz}\Phi^{pz};\;Y_{8}^{pz}=\frac{e^{pz}}{\varepsilon_{11}^{pz}}D_{3}^{pz}$$

(18) $$Y_{1}^{ice}=\frac{\omega }{V}C_{1111}^{pz}U_{1}^{ice};\;Y_{2}^{ice}=\frac{\omega }{V}C_{1111}^{pz}U_{2}^{ice};\;Y_{3}^{ice}=\frac{\omega }{V}C_{1111}^{pz}U_{3}^{ice};Y_{4}^{ice}=T_{13}^{ice};\;Y_{5}^{ice}=T_{23}^{ice};\;Y_{6}^{ice}=T_{33}^{ice}$$

(19) $$Y_{7}^{ice}=\frac{\omega }{V}e^{pz}\Phi^{ice};\;Y_{8}^{ice}=\frac{e^{pz}}{\varepsilon_{11}^{pz}}D_{3}^{ice}$$

(20) $$Y_{1}^{lq}=\frac{\omega }{V}C_{1111}^{pz}U_{1}^{ice};\;Y_{2}^{lq}=\frac{\omega }{V}C_{1111}^{pz}U_{2}^{lq};\;Y_{3}^{lq}=\frac{\omega }{V}C_{1111}^{pz}U_{3}^{lq};Y_{4}^{lq}=T_{13}^{lq};\;Y_{5}^{lq}=T_{23}^{lq};\;Y_{6}^{lq}=T_{33}^{lq}$$

(21) $$Y_{7}^{lq}=\frac{\omega }{V}e^{pz}\Phi^{lq};\;Y_{8}^{lq}=\frac{e^{pz}}{\varepsilon_{11}^{pz}}D_{3}^{lq}$$

(22) $$Y_{1}^{air}=\frac{\omega }{V}e^{pz}\Phi^{air};\;Y_{2}^{air}=\frac{e^{pz}}{\varepsilon_{11}^{pz}}D_{3}^{air}.$$

Here, *e^pz^* = 1 C/m^2^.

Expression (15) was substituted into Equations (1)–(14). At the same time, the variables *T*_11_, *T*_22_, *T*_12_, *D*_1_, and *D*_2_ were excluded from consideration for each medium, as they were not differentiated with respect to the x_3_ coordinate in Equations (1)–(14).

As a result, the systems of ordinary differential linear equations were obtained for each contacting media. For piezoelectric plates, ice, water and air, the numbers of these equations were 8, 8, 8, and 2, respectively. These systems further were re-written as:

(23) $$\left[dY_{j}^{pz}/dx_{3}\right]=\left[C^{pz}\right]Y_{j}^{pz}$$

(24) $$\left[dY_{j}^{ice}/dx_{3}\right]=\left[C^{ice}\right]Y_{j}^{ice}$$

(25) $$\left[dY_{j}^{lq}/dx_{3}\right]=\left[C^{lq}\right]Y_{j}^{lq}$$

(26) $$\left[dY_{j}^{air}/dx_{3}\right]=\left[C^{air}\right]Y_{j}^{air}$$

The matrices $\left[C^{pz}\right]$, $\left[C^{ice}\right]$, $\left[C^{lq}\right]$, and $\left[C^{air}\right]$ have dimensions of 8 × 8, 8 × 8, 8 × 8, and 2 × 2, respectively. The eigenvalues (*ζ_k_*) and the components of the eigenvectors (*Y_jk_*) of these matrices were obtained as the results of calculations.

The common solution for each medium was written as:

(27) $$Y_{j}^{pz}=\sum_{k=1}^{8}A_{k}^{pz}Y_{jk}^{pz}e^{\zeta_{j}^{pz}x_{3}}e^{iw(t-x_{1}/V)}$$

(28) $$Y_{j}^{ice}=\sum_{k=1}^{8}A_{k}^{ice}Y_{jk}^{ice}e^{\zeta_{j}^{ice}x_{3}}e^{iw(t-x_{1}/V)}$$

(29) $$Y_{j}^{lq}=\sum_{k=1}^{8}A_{k}^{lq}Y_{jk}^{lq}e^{\zeta_{j}^{lq}x_{3}}e^{iw(t-x_{1}/V)}$$

(30) $$Y_{j}^{air}=\sum_{k=1}^{2}A_{k}^{air}Y_{jk}^{air}e^{\zeta_{j}^{air}x_{3}}e^{iw(t-x_{1}/V)}$$

The weight coefficients $A_{k}^{pz}$, $A_{k}^{ice}$,$A_{k}^{lq}$, and $A_{k}^{air}$ as well phase velocity V are unknown values. To find them it is necessary to use the appropriate boundary conditions.

It should be noted that, in the case of plate contact with a half-infinite medium (ice, water, or air), it is necessary to make a choice of eigenvalues corresponding to the physical conditions of the problem. In all cases, the eigenvalues corresponding to the decrease in the electrical variables deep into the half-infinite medium were chosen. As for the mechanical variables, the eigenvalues corresponding to their increase deep into the half-infinite medium were selected. This choice was due to the need to take into account the radiation of acoustic wave energy deep into the contacting medium [58].

The electrical and mechanical conditions at boundaries *x*_3_ = 0 and *x*_3_ = *h* of each structure considered were used as follows [57,58]:

(31) $$x_{3}=0:\;T_{i3}^{pz}=0;\Phi^{pz}=\Phi^{air};D_{3}^{pz}=D_{3}^{air},$$

(32) $$x_{3}=h\;(\mathrm{contact}\;\mathrm{with}\;\mathrm{air}):\;T_{i3}^{pz}e^{\zeta^{pz}h}=0;\Phi^{pz}e^{\zeta^{pz}h}=\Phi^{air}e^{\zeta^{air}h};D_{3}^{pz}e^{\zeta^{pz}h}=D_{3}^{air}e^{\zeta^{air}h},$$

(33) $$x_{3}=h\;(\mathrm{contact}\;\mathrm{with}\;H_{2}O):\;U_{3}^{pz}e^{\zeta^{pz}h}=U_{3}^{lq}e^{\zeta^{H_{2}O}h};T_{33}^{pz}e^{\zeta^{pz}h}=T_{33}^{H_{2}O}e^{\zeta^{lq}h};T_{13}^{pz}e^{\zeta^{pz}h}=T_{23}^{pz}e^{\zeta^{pz}h}=0;$$

$$\Phi^{pz}e^{\zeta^{pz}h}=\Phi^{H_{2}O}e^{\zeta^{lq}h};D_{3}^{pz}e^{\zeta^{pz}h}=D_{3}^{lq}e^{\zeta^{lq}h}$$

(34) $$x_{3}=h\;(\mathrm{contact}\;\mathrm{with}\;\mathrm{ice}):\;U_{i}^{pz}e^{\zeta^{pz}h}=U_{i}^{ice}e^{\zeta^{ice}h};T_{i3}^{pz}e^{\zeta^{pz}h}=T_{i3}^{ice}e^{\zeta^{ice}h};\Phi^{pz}e^{\zeta^{pz}h}=\;\Phi^{ice}e^{\zeta^{ice}h};D_{3}^{pz}e^{\zeta^{pz}h}=D_{3}^{ice}e^{\zeta^{ice}h}.$$

Here, *i* = 1–3 and *h* is the thickness of a piezoelectric plate.

The matrix *D_apa_* of the boundary conditions for the structure “air–piezoelectric plate–air” (Figure 1a) with a dimension of 10 × 10 was obtained by using Equations (31) and (32).

airpiezoelectric plateair*x*_3_ = 00$Y_{41}^{pz}$$Y_{42}^{pz}$$Y_{43}^{pz}$$Y_{44}^{pz}$$Y_{45}^{pz}$$Y_{46}^{pz}$$Y_{47}^{pz}$$Y_{48}^{pz}$00$Y_{51}^{pz}$$Y_{52}^{pz}$$Y_{53}^{pz}$$Y_{54}^{pz}$$Y_{55}^{pz}$$Y_{56}^{pz}$$Y_{57}^{pz}$$Y_{58}^{pz}$00$Y_{61}^{pz}$$Y_{62}^{pz}$$Y_{63}^{pz}$$Y_{64}^{pz}$$Y_{65}^{pz}$$Y_{66}^{pz}$$Y_{67}^{pz}$$Y_{68}^{pz}$0$-Y_{11}^{air}$$Y_{71}^{pz}$$Y_{72}^{pz}$$Y_{73}^{pz}$$Y_{74}^{pz}$$Y_{75}^{pz}$$Y_{76}^{pz}$$Y_{77}^{pz}$$Y_{78}^{pz}$0*D_apa_* = ${-Y}_{21}^{air}$$Y_{81}^{pz}$$Y_{82}^{pz}$$Y_{83}^{pz}$$Y_{84}^{pz}$$Y_{85}^{pz}$$Y_{86}^{pz}$$Y_{87}^{pz}$$Y_{88}^{pz}$0*x*_3_ = *h*0$Y_{41}^{pz}e^{\zeta_{1}^{pz}h}$$Y_{42}^{pz}e^{\zeta_{2}^{pz}h}$$Y_{43}^{pz}e^{\zeta_{3}^{pz}h}$$Y_{44}^{pz}e^{\zeta_{4}^{pz}h}$$Y_{45}^{pz}e^{\zeta_{5}^{pz}h}$$Y_{46}^{pz}e^{\zeta_{6}^{pz}h}$$Y_{47}^{pz}e^{\zeta_{7}^{pz}h}$$Y_{48}^{pz}e^{\zeta_{8}^{pz}h}$00$Y_{51}^{pz}e^{\zeta_{1}^{pz}h}$$Y_{52}^{pz}e^{\zeta_{2}^{pz}h}$$Y_{53}^{pz}e^{\zeta_{3}^{pz}h}$$Y_{54}^{pz}e^{\zeta_{4}^{pz}h}$$Y_{55}^{pz}e^{\zeta_{5}^{pz}h}$$Y_{56}^{pz}e^{\zeta_{6}^{pz}h}$$Y_{57}^{pz}e^{\zeta_{7}^{pz}h}$$Y_{58}^{pz}e^{\zeta_{8}^{pz}h}$00$Y_{61}^{pz}e^{\zeta_{1}^{pz}h}$$Y_{62}^{pz}e^{\zeta_{2}^{pz}h}$$Y_{63}^{pz}e^{\zeta_{3}^{pz}h}$$Y_{64}^{pz}e^{\zeta_{4}^{pz}h}$$Y_{65}^{pz}e^{\zeta_{5}^{pz}h}$$Y_{66}^{pz}e^{\zeta_{6}^{pz}h}$$Y_{7}^{pz}e^{\zeta_{7}^{pz}h}$$Y_{68}^{pz}e^{\zeta_{8}^{pz}h}$00$Y_{71}^{pz}e^{\zeta_{1}^{pz}h}$$Y_{72}^{pz}e^{\zeta_{2}^{pz}h}$$Y_{73}^{pz}e^{\zeta_{3}^{pz}h}$$Y_{74}^{pz}e^{\zeta_{4}^{pz}h}$$Y_{75}^{pz}e^{\zeta_{5}^{pz}h}$$Y_{76}^{pz}e^{\zeta_{6}^{pz}h}$$Y_{77}^{pz}e^{\zeta_{7}^{pz}h}$$Y_{78}^{pz}e^{\zeta_{8}^{pz}h}$${-Y}_{11}^{air}e^{\zeta_{1}^{air}h}$0$Y_{81}^{pz}e^{\zeta_{1}^{pz}h}$$Y_{82}^{pz}e^{\zeta_{2}^{pz}h}$$Y_{83}^{pz}e^{\zeta_{3}^{pz}h}$$Y_{84}^{pz}e^{\zeta_{4}^{pz}h}$$Y_{85}^{pz}e^{\zeta_{5}^{pz}h}$$Y_{86}^{pz}e^{\zeta_{6}^{pz}h}$$Y_{87}^{pz}e^{\zeta_{7}^{pz}h}$$Y_{88}^{pz}e^{\zeta_{8}^{pz}h}$${-Y}_{21}^{air}e^{\zeta_{1}^{air}h}$

The matrix *D_apl_* of the boundary conditions for the structure “air–piezoelectric plate–distilled water” (Figure 1b) with dimension of 11 × 11 was obtained by using Equations (31) and (33).

airpiezoelectric platehalf-infinite *H_2_O**x*_3_ = 00$Y_{41}^{pz}$…$Y_{48}^{pz}$000$Y_{51}^{pz}$…$Y_{58}^{pz}$000$Y_{61}^{pz}$…$Y_{68}^{pz}$00$-Y_{11}^{air}$$Y_{71}^{pz}$…$Y_{78}^{pz}$00${-Y}_{21}^{air}$$Y_{81}^{pz}$…$Y_{88}^{pz}$00*D_apl_* =0$Y_{31}^{pz}e^{\zeta_{1}^{pz}h}$…$Y_{38}^{pz}e^{\zeta_{8}^{pz}h}$${-Y}_{31}^{lq}e^{\zeta_{1}^{lq}h}$0*x*_3_ = *h*0$Y_{41}^{pz}e^{\zeta_{1}^{pz}h}$…$Y_{48}^{pz}e^{\zeta_{8}^{pz}h}$000$Y_{51}^{pz}e^{\zeta_{1}^{pz}h}$…$Y_{58}^{pz}e^{\zeta_{8}^{pz}h}$000$Y_{61}^{pz}e^{\zeta_{1}^{pz}h}$…$Y_{68}^{pz}e^{\zeta_{8}^{pz}h}$${-Y}_{61}^{lq}e^{\zeta_{1}^{lq}h}$00$Y_{71}^{pz}e^{\zeta_{1}^{pz}h}$…$Y_{78}^{pz}e^{\zeta_{8}^{pz}h}$0${-Y}_{71}^{lq}e^{\zeta_{1}^{lq}h}$0$Y_{81}^{pz}e^{\zeta_{1}^{pz}h}$…$Y_{88}^{pz}e^{\zeta_{8}^{pz}h}$0${-Y}_{81}^{lq}e^{\zeta_{1}^{lq}h}$

The matrix *D_api_* of the boundary conditions for the structure “air–piezoelectric plate–ice” (Figure 1c) with dimension of 13 × 13 was obtained by using Equations (31) and (34).

airpiezoelectric platehalf-infinite ice*x*_3_ = 00$Y_{41}^{pz}$…$Y_{48}^{pz}$00000$Y_{51}^{pz}$…$Y_{58}^{pz}$00000$Y_{61}^{pz}$…$Y_{68}^{pz}$0000$-Y_{11}^{air}$$Y_{71}^{pz}$…$Y_{78}^{pz}$0000${-Y}_{21}^{air}$$Y_{81}^{pz}$…$Y_{88}^{pz}$0000*D_api_* = 0$Y_{11}^{pz}e^{\zeta_{1}^{pz}h}$…$Y_{18}^{pz}e^{\zeta_{8}^{pz}h}$${-Y}_{11}^{ice}e^{\zeta_{1}^{ice}h}$${-Y}_{12}^{ice}e^{\zeta_{2}^{ice}h}$${-Y}_{13}^{ice}e^{\zeta_{3}^{ice}h}$0*x*_3_ = *h*0$Y_{11}^{pz}e^{\zeta_{1}^{pz}h}$…$Y_{28}^{pz}e^{\zeta_{8}^{pz}h}$${-Y}_{21}^{ice}e^{\zeta_{1}^{ice}h}$${-Y}_{22}^{ice}e^{\zeta_{2}^{ice}h}$${-Y}_{23}^{ice}e^{\zeta_{3}^{ice}h}$00$Y_{31}^{pz}e^{\zeta_{1}^{pz}h}$…$Y_{38}^{pz}e^{\zeta_{8}^{pz}h}$${-Y}_{31}^{ice}e^{\zeta_{1}^{ice}h}$${-Y}_{32}^{ice}e^{\zeta_{2}^{ice}h}$${-Y}_{33}^{ice}e^{\zeta_{3}^{ice}h}$00$Y_{41}^{pz}e^{\zeta_{1}^{pz}h}$…$Y_{48}^{pz}e^{\zeta_{8}^{pz}h}$${-Y}_{41}^{ice}e^{\zeta_{1}^{ice}h}$${-Y}_{42}^{ice}e^{\zeta_{2}^{ice}h}$${-Y}_{43}^{ice}e^{\zeta_{3}^{ice}h}$00$Y_{51}^{pz}e^{\zeta_{1}^{pz}h}$…$Y_{58}^{pz}e^{\zeta_{8}^{pz}h}$${-Y}_{51}^{ice}e^{\zeta_{1}^{ice}h}$${-Y}_{52}^{ice}e^{\zeta_{2}^{ice}h}$${-Y}_{53}^{ice}e^{\zeta_{3}^{ice}h}$00$Y_{61}^{pz}e^{\zeta_{1}^{pz}h}$…$Y_{68}^{pz}e^{\zeta_{8}^{pz}h}$${-Y}_{61}^{ice}e^{\zeta_{1}^{ice}h}$${-Y}_{62}^{ice}e^{\zeta_{2}^{ice}h}$${-Y}_{63}^{ice}e^{\zeta_{3}^{ice}h}$00$Y_{71}^{pz}e^{\zeta_{1}^{pz}h}$…$Y_{78}^{pz}e^{\zeta_{8}^{pz}h}$000${-Y}_{71}^{ice}e^{\zeta_{1}^{ice}h}$0$Y_{81}^{pz}e^{\zeta_{1}^{pz}h}$…$Y_{88}^{pz}e^{\zeta_{8}^{pz}h}$000${-Y}_{81}^{ice}e^{\zeta_{1}^{ice}h}$

The phase velocity *V* of the acoustic wave is a parameter of the recorded matrices of boundary conditions [*D_apa_*], [*D_apl_*], or [*D_api_*]. The values of phase velocity corresponding to each boundary task (Figure 1) were determined using an iterative search procedure based on zeroing the determinant of the matrices [*D_apa_*], [*D_apl_*], or [*D_api_*], respectively [56]. After that, the weight coefficients $A_{k}^{pz}$, $A_{k}^{air}$, $A_{k}^{ice}$, or $A_{k}^{lq}$ were calculated by using the corresponding boundary condition matrix. Then, three partial components of mechanical displacement (*U*_1_, *U*_2_, *U*_3_) in the plane *x*_3_ = *h* were defined by using Equation (27).

The material constants for *LiNbO*_3_, *LiTaO*_3_, water at T = 20 °C, and ice at T = −15 °C were taken from [59,60,61,62]. All material constants used in calculation are presented in Table 1.

It should be noted that the effect of low temperature (−15 °C) on the properties of the piezoelectric plate was taken into account by using the procedure described in [63]. As a result, it was found that at this temperature, the velocity of the acoustic waves increase insignificantly by 0.5–1% and the attenuation remains practically unchanged. Therefore, the results obtained without considering the influence of temperature on the material constants of the piezoelectric plates were used in this study.

The attenuation of acoustic waves (Γ and dB/λ, where λ is the wavelength) propagating in piezoelectric plates contacting with half-space ice or liquid is attributed with radiation losses of acoustic energy deep into these media. The value of such attenuation was calculated as:

(35) $$\Gamma =54.51\times V_{im}/V_{re},$$

where *V_im_* and *V_re_* are the image and real parts of the phase velocity, respectively.

In accordance with the experimental data, calculations were carried out for higher-order Lamb and QSH acoustic waves in the 128YX+*Θ LiNbO*_3_ plate (*h*/*λ* = 2.5), YX+*Θ LiNbO*_3_ plate (*h*/*λ* = 2.5), and 36YX+*Θ LiTaO*_3_ plate (*h*/*λ* = 1.75) at frequencies ranging from 20 to 60 MHz and propagation angles of *Θ* = 0°, 30°, 60°, and 90_°_ to the *X*-axis.

As a result of the calculations, the electromechanical coupling coefficient (*k*^2^,%) [64] of the considered waves and their mechanical displacements on the surface of the piezoelectric plate (*x*_3_ = *h*), normalized by the longitudinal component *U*_1_, were also determined.

*k*^2^ = 200 × (*V* − *V_m_*)/*V*, (36)

where *V_m_* is the phase velocity of the acoustic wave in a plate metalized from one side.

The identification of the wave was based on the similarity between the calculated and measured values of its frequency.

### 2.2. Fabrication of Devices with Anisotropic Propagation of Acoustic Waves

Three samples of experimental devices were created using the plates 128Y-cut *LiNbO*_3_, Y-cut *LiNbO*_3_ (h = 500 μm), and 36Y-cut *LiTaO*_3_ (h = 350 μm). The piezoelectric plates were purchased at CQT (Hangzhou, China). On the polished face of each wafer, four delay lines were positioned in a circular arrangement at angles of 0°, 30°, 60°, and 90° to the *X*-axis. These angles were selected based on the maximum anisotropy of this crystallographic orientation, as reported in [64,65].

The delay lines on the surface of the experimental devices were produced by using the photolithography procedure described in detail in [56].

**Figure 2:**
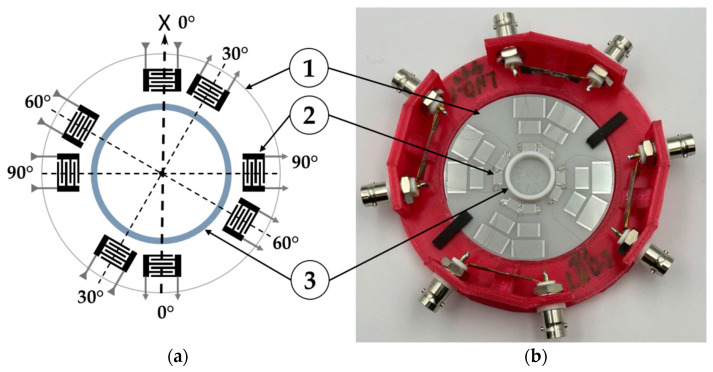
(**a**) Schematic view and (**b**) photo of an experimental sample with a set of acoustic delay lines (or channels) on a single piezoelectric wafer. The acoustic channels are oriented at angles *Θ* = 0°, 30°, 60°, and 90° with respect to the crystallographic *X*-axis. 1—piezoelectric plate, 2—IDT, 3—the cell with a diameter of 18 mm.

Figure 2 shows a schematic image (a) and a photograph (b) of an experimental setup with a set of acoustic delay lines (or channels) on a single piezoelectric crystal. During the experiments, only the first ring of the delay lines (closest to the cell) was used (Figure 2).

The input and output interdigital transducers (IDTs) used had a period of *λ* = 200 μm and the same topology. In the center of a wafer, a cell with diameter of 18 mm was glued. The analyzed substance was placed into this cell during the experiments.

It should be noted that, due to the anisotropy of piezoelectric plates, the energy flows of most waves traveling in directions with low symmetry (*Θ* = 30° and 60°) do not align with the propagation direction [65]. However, the magnitude of the deflection angles of the energy flows was small (<5°), which made it possible to detect the excited waves using output IDTs placed on the opposite side of a cell.

### 2.3. Measurement Technique

**Figure 3:**
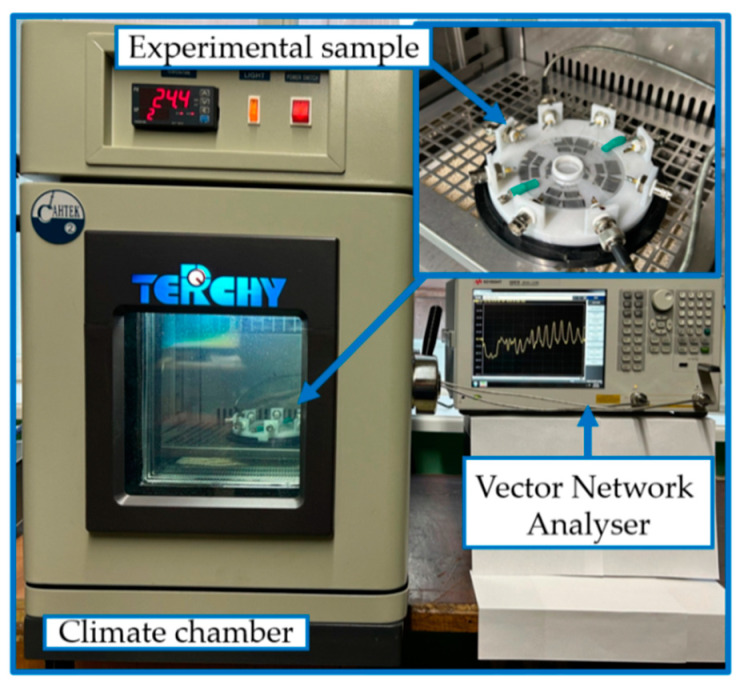
The experimental setup and sample used.

The measurements of the insertion loss (*S*_12_) of each delay line were taken in the frequency range of *f* = 3–60 MHz under an atmospheric pressure of *p* = 746 mm Hg and at temperatures T = +20 °C and −15 °C. The *S*_12_ versus frequency *f* was measured using a network analyzer E5061B (Keysight, SantaRosa, CA, USA) with an accuracy of less than ±0.1 dB. It operated in the amplitude–frequency format *S*_12_(*f*). To measure S_12_ at different temperatures, the experimental sample was placed into a climatic chamber UC-20CE (Terchy, Nantou, Taiwan). This chamber was operated in the temperature range of *T* = −60 °C to +50 °C. The experimental setup is shown in Figure 3.

Initially, the frequency dependence of $S_{12}^{air\;}$ was measured for a piezoelectric plate without loading at a temperature of *T* = 20 °C. After that, distilled water was placed in the cell and measurements of the frequency dependence of $S_{12}^{lq\;}$ were repeated at a temperature of *T* = 20 °C. At this point, the viscosity of the liquid η was 1.03 cP, and its conductivity σ was below 0.001 S/m. Then, the temperature was dropped to −15 °C, while the liquid turned into ice, and the signal started to change. After the signal stabilized, measurements of the frequency dependence of $S_{12}^{ice}$ were repeated with the ice present. The acoustic response to the phase transition of matter from one state to another was represented by the value $∆S_{12}(dB)={|S}_{12}^{ice}|-|S_{12}^{lq}|$. This parameter is used to compare the effect of the phase transition on Lamb and QSH waves with different propagation directions in various materials. Due to the low penetration depth of the acoustic fields into liquid (0.1 μm) compared to its thickness (>1 mm), the shape and thickness of the test sample had no significant effect on the measurement results. In our experiments, liquid samples with a volume of 500 μL were used.

## 3. Results and Discussion

### 3.1. Experimental Results

The experimental study of the influence of the water-to-ice phase transition on the properties of Lamb and QSH acoustic waves of different orders (or numbers *n*), depending on their propagation direction, were carried out using the experimental samples (Figure 2) and experimental setup (Figure 3). The acoustic waves under study experienced different levels of acoustic absorption depending on the propagation direction. This is because in an anisotropic plate the sensitivity of each wave to the physical parameters of the liquid or ice is different.

**Figure 4:**
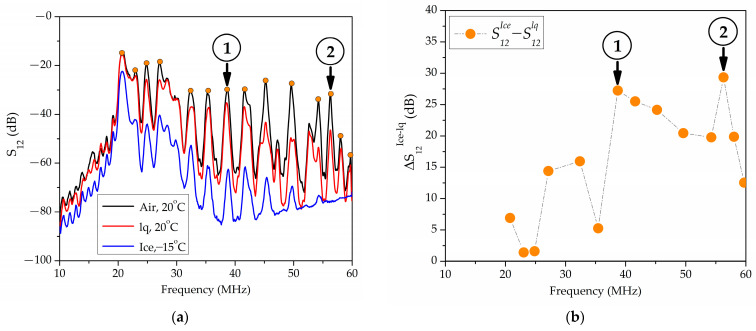
The frequency dependences of (**a**) the insertion losses *S*_12_ and (**b**) the responses $∆S_{12}^{ice-lq}$, measured in a 36°YX+*Θ LiTaO*_3_ plate at h/λ = 2.5, *Θ* = 0° in the absence of a load (+20 °C, black line), with distilled water (+20 °C, red line), and with ice (−15 °C, blue line) in the cell. Waves with the highest values of $∆S_{12}^{ice-lq}$: *f* = 36.69 MHz, $∆S_{12}^{ice-lq}$ = 27.2 dB (1), *f* = 56.69 MHz, and $∆S_{12}^{ice-lq}$ = 29.3 dB (2).

Experiments have shown that the transition from water to ice is accompanied by an increase in insertional losses *S*_12_ and a positive response value $∆S_{12}^{ice-lq}$. The value $∆S_{12}^{ice-lq}$ depends on the propagation direction, wave frequency, thickness, and material of a plate. Figure 4 shows the typical frequency dependence of the insertion loss *S*_12_ (a) and responses $∆S_{12}^{ice-lq}$ (b) measured in a 36°YX+*Θ LiTaO*_3_ plate with a thickness of *h* = 500 μm, *λ* = 200 μm, *h*/*λ* = 2.5, and *Θ* = 0° in the absence of a load (+20 °C, black line), in the presence of distilled water (+20 °C, red line), and in the presence of ice (−15 °C, blue line) in a cell. In Figure 4, the arrows indicate the waves with the maximum values of $∆S_{12}^{ice-lq}$: f = 36.69 MHz, $∆S_{12}^{ice-lq}\;$= 27.2 dB (1), f = 56.69 MHz, and $∆S_{12}^{ice-lq}$ = 29.3 dB (2).

An analysis of the experimental data revealed that the maximum values of the $∆S_{12}^{ice-lq}$ in different piezoelectric plates and acoustic channels varied widely (14–42 dB). The minimum values of $∆S_{12}^{ice-lq}$ lie in the range of 0.87–3 dB. This means that in each plate, depending on its crystallographic orientation, there are both acoustic waves with a high sensitivity to the phase transitions and waves that are almost insensitive to them.

The maximum values of $∆S_{12}^{ice-lq}$ experimentally measured for acoustic waves in the 128YX+*Θ LiNbO*_3_ (*h*/*λ* = 2.5), YX+*Θ LiNbO*_3_ (*h*/*λ* = 2.5), and 36YX+*Θ LiTaO*_3_ (*h*/*λ* = 1.75) plates, in the frequency range of 20–60 MHz, for propagation direction angles *Θ* = 0°, 30°, 60°, 90°, as well as their corresponding operating frequencies, are shown in Table 2.

**Figure 5:**
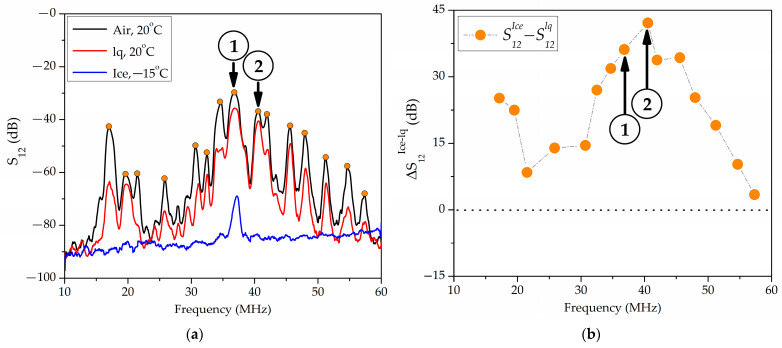
The frequency dependences of (**a**) the insertion losses *S*_12_ and (**b**) the responses $∆S_{12}^{ice-lq}$, measured in a YX+30° *LiNbO*_3_ plate with *h*/*λ* = 1.75. Wave (1) has *f* = 36.8 MHz {1; 3; 0.7}, and $∆S_{12}^{ice-lq}$ = 36.2 dB. Wave (2) has *f* = 40.53 MHz {1, 0, 0.3}, and $∆S_{12}^{ice-lq}$ = 42.1 dB.

In addition, unusual combinations of the plate material, plate thickness, and propagation direction were experimentally detected (Figure 5). For these combinations, the formation of ice led to the complete absorption of all waves excluding one (Figure 5a, *f* = 36.8 MHz) wave.

It should be noted that the elastic polarizations of the acoustic waves depend on the direction of propagation (angle Θ), the plate thickness *h*/*λ*, and the wave number n. At fixed *Θ*, *h*/*λ,* and *n* the polarizations include the entire spectrum of known Lamb and QSH waves; namely, oscillations along an elliptical path, in which the longitudinal, horizontal shear, and vertical displacements are approximately equal (*U*_1_~*U*_2_~*U*_3_); horizontal shear oscillations (*U*_2_ >> *U*_1_, *U*_3_); quasi-longitudinal oscillations (*U*_1_ >> *U*_2_, *U*_3_), as well as oscillations along an ellipse parallel to the surfaces of the plate (*U*_1_~*U*_2_, *U*_3_ ≈ 0); and an ellipse perpendicular to both the surfaces of the plate and the direction of propagation (*U*_2_~*U*_3_, *U*_1_ ≈ 0). It is clear that the physical causes of the observed anomaly are attributed to the anisotropy of the plates, the properties of the acoustic waves, and the characteristics of the load. In order to better understand the behavior of these waves in both unloaded and loaded plates, a theoretical analysis was carried out.

### 3.2. Theoretical Results

As a result of solving the system of Equations (1)–(4), (13), and (14), taking into account the boundary conditions (31) and (32), the values of the phase velocities ($V_{air}^{th}$) of the acoustic waves, their electromechanical coupling coefficients (*k*^2^), and polarization (*U*_1_, *U*_2_, *U*_3_) in the frequency range of interest, for propagation angles *Θ* = 0°, 30°, 60°, 90° in the 128YX+*Θ LiNbO*_3_ (*h*/*λ* = 2.5), YX+*Θ LiNbO*_3_ (*h*/*λ* = 2.5), and 36YX+*Θ LiTaO*_3_ (*h*/*λ* = 1.75) plates, were calculated. The results obtained were used to identify piezoactive Lamb and QSH acoustic waves that can be excited under these conditions.

Further, by using the system of Equations (1)–(4) and (9)–(14), and taking into account the boundary conditions (31) and (33), the problem of the propagation of acoustic waves in the structure “air–piezoelectric plate–semi-infinite water” was solved. The initial wave velocities in the calculations were based on the results obtained for plates without a load. As a result, the phase velocity ($V_{lq}^{th}$) and the attenuation ($\Gamma_{lq}^{th}$) of acoustic waves in such structures, as well their polarization in the plane *x*_3_ = *h*, were obtained.

At the next stage, the problem of acoustic wave propagation in the structure “air–piezoelectric plate–semi-infinite ice” was solved. For this purpose, the system of Equations (1)–(8), (13), and (14) was used, taking into account the boundary conditions (31) and (34). As a result, the values of the phase velocities of the acoustic waves ($V_{ice}^{th}$) and their attenuation ($\Gamma_{ice}^{th}$) and polarization in the *x*_3_ = *h* plane were also obtained.

The calculated values of the phase velocities, electromechanical coupling coefficients, polarizations, and maximum attenuation responses to glaciation for the experimentally determined frequencies of the acoustic waves are shown in Table 3, Table 4 and Table 5. It should be noted that the theoretically calculated acoustic wave frequencies differ from the experimentally measured ones (Table 2) by 1–2 MHz. This discrepancy is due to the difference between the values of material constants for solid media obtained from the literature and those used in the experiment with actual materials.

The analysis of the numerical results revealed that the phase velocity of acoustic waves does not significantly change in the presence of liquid or ice. Moreover, the polarization of the waves under study remains unchanged in the presence of the load. This can be attributed to the low acoustic impedance of water and ice in comparison to piezoelectric materials. It should be noted that the attenuation of acoustic waves occurs in the presence of a load, due to the radiation losses of acoustic energy during wave propagation. These losses are generally greater for ice than for water, which is consistent with the experimental data [44]. Additionally, the analysis showed that the value of attenuation theoretically calculated is lower than the experimentally measured ones (Table 2). This is because the theoretical analysis does not take into account the losses in IDT for converting an electrical signal into an acoustic signal and vice versa. It also does not consider losses due to the scattering of acoustic waves on ice defects.

It was also found that the change in attenuation due to the water-to-ice phase transition depends on the material of the plate. For example, for lithium tantalate, the magnitude of these changes is smaller than for lithium niobate. This is because although these two materials belong to the same crystallographic class and have a similar type of anisotropy, their densities differ by almost twice. This increases the impedance difference between the plate and loading.

The analysis also showed that the waves with a large *U*_2_ component have less sensitivity to phase transitions than waves with the largest *U*_1_ or *U*_3_ components. For example, the waves in a YX+30° *LiNbO*_3_ plate at f = 40.53 MHz {1, 0, 0.3} with $∆S_{12}^{ice-lq}$ = 42.1 dB and in a YX+60° *LiNbO*_3_ plate at *f* = 56.5 MHz {1, 3.2, 0.8} with $∆S_{12}^{ice-lq}$ = 0.87 dB are the most and least sensitive to the phase transition, respectively.

Therefore, when designing devices for detecting the water-to-ice phase transition, it may be advisable to use the acoustic waves with the maximum components of *U*_1_ and *U*_3_.

## 4. Conclusions

This theoretical and experimental study of the effect of water-to-ice phase transitions on the properties of higher-order Lamb and QSH waves propagating in various directions through anisotropic piezoelectric plates has shown that the effect of the anisotropy of Lamb and QSH waves on the detection of the liquid-to-ice phase transition is evident in their different absorptions during propagation in various directions. The theoretical calculations and experiments have shown that the transition from water to ice is accompanied by an increase in insertional losses *S*_12_ and a positive response value $∆S_{12}^{ice-lq}$. The experimental absorption response values of $∆S_{12}^{ice-lq}$ range from 0.87 dB to 42.1 dB, depending on the propagation direction, wave type, wave thickness, and the material of the plate. This means that in each plate depending on its crystallographic orientation there are both acoustic waves with a high sensitivity to the phase transitions and waves that are almost insensitive to them.

During the experiments, an interesting result was obtained. It was found that in a YX+30° *LiNbO*_3_ plate with *h*/*λ* = 1.75, the formation of ice led to the complete absorption of all waves excluding one. The theoretical analysis showed that this wave has quasi-shear horizontal polarization and does not completely attenuate due to glaciation.

In conclusion, the use of materials with higher anisotropy in their acoustic properties can lead to the development of more sensitive sensors for the detection and analysis of liquid–solid phase transitions.

Acoustic waves with a high sensitivity to liquid–solid phase transition can be used to create glaciation sensors, and weakly sensitive waves can be useful for other sensors operating under conditions of a change in the aggregate state of the analyzed substance.

## Figures and Tables

**Table 1:** Density *ρ* (kg/m^3^), elastic constants *C_ij_* (GPa), piezoelectric coefficients *e_ij_* (C/m^2^), and dielectric permittivity *ε_ij_*/*ε*_0_ of *LiNbO*_3_, *LiTaO*_3_, *ice*, and nonviscous, nonconducting distilled water used in calculations.

*LiNbO*_3_						
$C_{11}^{E}$	$C_{12}^{E}$	$C_{13}^{E}$	$C_{14}^{E}$	$C_{33}^{E}$	$C_{44}^{E}$	$C_{66}^{E}$
203	57.3	75.2	8.5104	242.4	59.5	72.8
*e*_15_	*e*_16_	*e*_31_	*e*_33_	*ε*_11_/*ε*_0_	*ε*_33_/*ε*_0_	*ρ*
3.84	−2.37	0.23	1.3	44.305	27.9	4650
***LiTaO*_3_**						
$C_{11}^{E}$	$C_{12}^{E}$	$C_{13}^{E}$	$C_{14}^{E}$	$C_{33}^{E}$	$C_{44}^{E}$	$C_{66}^{E}$
232.8	46.5	83.6	−10.5	275.9	94.9	93.15
*e*_15_	*e*_16_	*e*_31_	*e*_33_	*ε*_11_/*ε*_0_	*ε*_33_/*ε*_0_	*ρ*
2.64	−1.68	−0.22	1.71	40.904	42.5	7454
**Ice** (at *T* = −15 °C)						
$C_{11}^{E}$	$C_{33}^{E}$	$C_{12}^{E}$	$C_{13}^{E}$	$C_{44}^{E}$	*ε*_11_/*ε*_0_	*ρ*
13.9	14.94	6.749	5.701	2.917	3.26	900
$H_{2}O\;(\mathrm{at}T\;=20\;{}^{\circ}C):\;\rho =997.299;\;C_{11}^{E}$ = 2.25; *ε*_11_/*ε*_0_ = 80						

**Table 2:** Experimentally obtained maximum responses $∆S_{12}^{ice-lq}$ for the water-to-ice phase transition in acoustic waves in the YX+*Θ LiNbO*_3_, 128Y X+*Θ LiNbO*_3_, and 36Y X+*Θ LiTaO*_3_ piezoelectric plates at different propagation directions and the corresponding operating frequencies.

YX+*Θ LiNbO*_3_ plate, *h* = 350 μm, *λ* = 200 μm, *h*/*λ* = 1.75							
***Θ* = 0°**		***Θ* = 30°**		***Θ* = 60°**		***Θ* = 90°**	
*f*, MHz	$∆S_{12}^{ice-lq}$, dB	*f*, MHz	$∆S_{12}^{ice-lq}$ dB	*f*, MHz	$∆S_{12}^{ice-lq}$, dB	*f*, MHz	$∆S_{12}^{ice-lq}$, dB
42.1	31.9	36.8	36.2	21.59	28.3	25.38	32.2
45.6	31.8	40.53	42.1	36.34	37.5	34.28	26.5
48.1	28.7	45.56	34.9	40.25	38.6	44.34	28.2
**128YX+*Θ LiNbO*_3_ plate, *h* = 500 μm, *λ* = 200 μm, *h*/*λ* = 2.5**							
*f*, MHz	$∆S_{12}^{ice-lq}$, dB	*f*, MHz	$∆S_{12}^{ice-lq}$ dB	*f*, MHz	$∆S_{12}^{ice-lq}$, dB	*f*, MHz	$∆S_{12}^{ice-lq}$, dB
26.5	23.6	24.66	23.9	33.25	27.6	29.1	14.4
46.22	21.4	34.78	26	35.08	28.5	38.06	18.8
50.16	22	38.06	26	37.84	30.6	41.5	20.6
**36YX+*Θ LiTaO*_3_ plate, *h* = 500 μm, *λ* = 200 μm, *h*/*λ* = 2.5**							
*f*, MHz	$∆S_{12}^{ice-lq}$, dB	*f*, MHz	$∆S_{12}^{ice-lq}$ dB	*f*, MHz	$∆S_{12}^{ice-lq}$, dB	*f*, MHz	$∆S_{12}^{ice-lq}$, dB
38.69	27.2	43.78	28.25	21.25	24.5	41.25	25.8
41.59	25.5	52.72	29.4	44.13	24.3	43.16	25.1
56.28	29.3	58.69	27.1	52.66	27.7	48.69	27.1

**Table 3:** The theoretically obtained parameters of acoustic waves in the structures “air–YX+Θ-*LiNbO*_3_ plate with *h*/λ = 1.75–air”, “air–YX+Θ-*LiNbO*_3_ plate with *h*/λ = 1.75–water”, and “air–YX+Θ-*LiNbO*_3_ plate with *h*/λ = 1.75–ice”.

*f*^th^, MHz	$V_{air}^{th}$, km/s	*k*^2^, %	*U*_1_; *U*_2_; *U*_3_	$V_{lq}^{th}$, km/s	$V_{ice}^{th}$, km/s	${\Delta \Gamma }_{ice-lq}^{th}$, dB
***Θ* = 0°**						
40.8	14.288	3.9	1; 12; 1	14.260	14.286	24.8
42.7	14.954	0.05	1; 0.1; 2	14.954	14.969	26.0
44.1	15.425	5.6	1; 0; 0.2	15.399	15.424	26.6
47.5	16.641	2.4	1; 15; 2.4	16.622	16.639	21.5
***Θ* = 30°**						
36.5	12.753	4.6	1; 3; 0.7	12.722	12.751	27.8
40.5	14.201	3.6	1; 0; 0.3	14.181	14.199	29.4
44.2	15.458	5.1	1; 0.2; 0.1	15.431	15.456	26.8
***Θ* = 60°**						
21.5	7.517	2.0	1; 0.1; 0.4	7.474	7.512	50.2
36.9	12.932	1.5	1; 0.4; 0.4	12.922	12.931	31.6
40.4	14.138	1.8	1; 0.7; 0.1	14.127	14.137	28.4
***Θ* = 90°**						
26.4	9.251	1.4	1; 0; 0.7	9.234	9.249	42.5
33.5	11.732	1.6	1; 0; 0.6	11.718	11.731	34.6
44.6	15.633	7.2	1; 0; 0.4	15.597	15.630	26

**Table 4:** The theoretically obtained parameters of acoustic waves in the structure “air–128YX+Θ *LiNbO*_3_ plate with *h*/λ = 2.5–air”, “air–128YX+Θ *LiNbO*_3_ plate with *h*/λ = 2.5–water”, “air–128YX+Θ *LiNbO*_3_ plate with *h*/λ = 2.5–ice”.

*f*^th^, MHz	$V_{air}^{th}$, km/s	*k*^2^, %	*U*_1_; *U*_2_; *U*_3_	$V_{lq}^{th}$, km/s	$V_{ice}^{th}$, km/s	${\Delta \Gamma }_{ice-lq}^{th}$, dB
***Θ* = 0°**						
26.3	13.140	1.3	1; 0.1; 0.3	13.129	13.139	19.3
47.4	23.747	2.3	1; 0; 0.3	23.736	23.746	11.2
50.3	25.164	4.4	1; 0; 0.1	25.142	25.163	10.1
***Θ* = 30°**						
25.2	12.619	2.9	1; 0.7; 1	12.594	12.618	17.5
34.0	17.001	3.6	1; 0.5; 0.3	16.980	17.001	15.0
37.4	18.711	2.0	1; 2.8; 5.1	18.697	18.711	13.3
***Θ* = 60°**						
33.4	16.703	1.5	1; 0.6; 0.2	16.697	16.703	16.7
34.2	17.093	0.8	1; 0.9; 2.6	17.088	17.092	15.2
37.1	18.537	0.9	1; 5.5; 7.4	18.531	18.536	14.1
***Θ* = 90°**						
29.4	14.702	1.0	1; 0; 1.4	14.694	14.701	17.4
37.2	18.568	1.1	1; 0; 1.8	18.560	18.567	14.3
41.2	20.597	0.4	1; 0; 0.2	20.595	20.597	13.6

**Table 5:** The theoretically obtained parameters of acoustic waves in the structures “air–36YX+Θ *LiTaO*_3_ plate with *h*/λ = 2.5–air”, “air–36YX+Θ *LiTaO*_3_ plate with *h*/λ = 2.5–water”, “air–36YX+Θ *LiTaO*_3_ plate with *h*/λ = 2.5–ice”.

*f*^th^, MHz	$V_{air}^{th}$, km/s	*k*^2^, %	*U*_1_; *U*_2_; *U*_3_	$V_{lq}^{th}$, km/s	$V_{ice}^{th}$, km/s	${\Delta \Gamma }_{ice-lq}^{th}$, dB
***Θ* = 0°**						
39.1	19.555	1.3	1; 0.1; 1.6	19.550	19.555	8.9
41.4	20.719	2.1	1; 2.6; 40	20.713	20.719	8.6
56.4	28.222	2.2	1; 1.4; 5.3	28.217	28.221	6.4
***Θ* = 30°**						
34.0	17.001	3.6	1; 0.5; 0.3	16.980	17.001	7.9
37.4	18.711	2.0	1; 2.8; 5.1	18.697	18.711	7.0
43.8	21.901	0.9	1; 0.2; 1	21.899	21.901	7.9
***Θ* = 60°**						
21.3	10.627	0.2	1; 0.8; 0.4	10.626	10.626	16.4
44.3	22.145	2.0	1; 0.6; 2.5	22.138	22.144	7.9
51.6	25.818	2.1	1; 0.5; 4.8	25.812	25.817	6.9
***Θ* = 90°**						
41.7	20.871	1.7	1; 0; 2.6	20.865	20.871	8.3
42.4	21.210	0.9	1; 0; 0.4	21.207	21.210	8.1
47.6	23.800	0.6	1; 0; 0.2	23.798	23.800	7.1

## Data Availability

Data are contained within the article.
